# 3-(4-Chloro­phen­yl)-3-hy­droxy-1-phenyl­prop-2-ene-1-thione

**DOI:** 10.1107/S2414314626005638

**Published:** 2026-06-02

**Authors:** A S Jeevan Chakravarthy, N R Sreenatha, Nagesh Babu

**Affiliations:** a Department of Chemistry BMS Institute of Technology & Management Autonomous under Visvesvaraya Technological University, Avalahalli Yelahanka Bengaluru Karnataka 560064 India; b Department of Physics, Government Engineering College, Bedarapura, Chamarajanagara, Karnataka 571313, India; c Department of Biochemistry Maharani Cluster University, Bangalore 560001 Karnataka India; University of Aberdeen, United Kingdom

**Keywords:** chloro­phen­yl, thioxopropan, dihedral angle, crystal structure

## Abstract

In the title compound, C_15_H_11_ClOS, the dihedral angle between the aromatic rings is 13.06 (6)° and an intra­molecular O—H⋯S hydrogen bond supports the mol­ecular conformation. In the extended structure, weak C—H⋯O hydrogen bonds connect the mol­ecules into [010] chains.

## Abstract 2

In the title compound, the dihedral angle between the aromatic rings is 13.06 (6)° and an intra­molecular O—H⋯S hydrogen bond supports the mol­ecular conformation. In the extended structure, weak C—H⋯O hydrogen bonds connect the mol­ecules into [010] chains.

## Graphical abstract

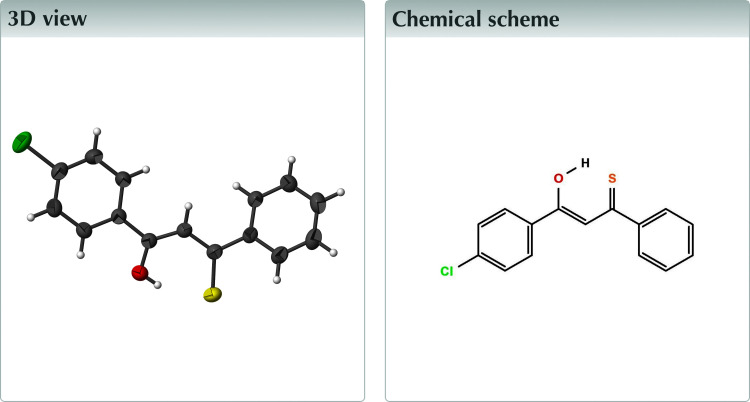

## Structure description

**Figure 1:**
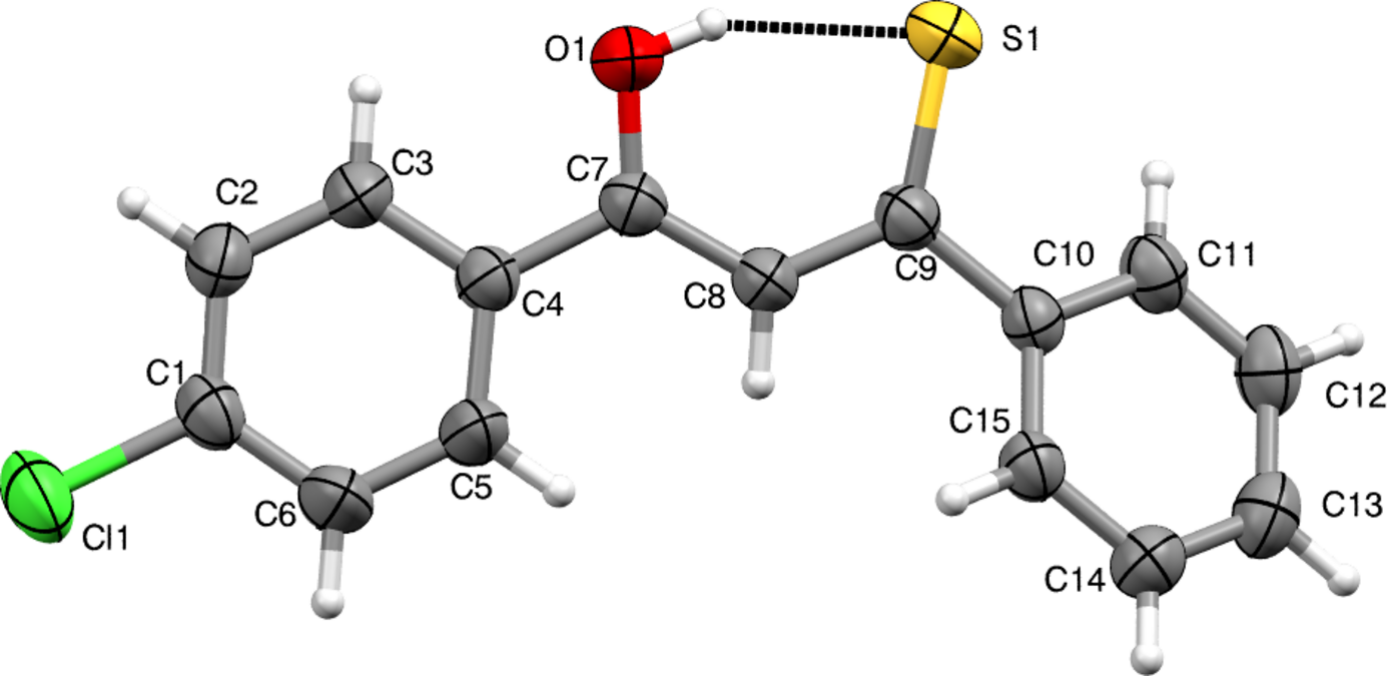
The mol­ecular structure of the title compound with displacement ellipsoids drawn at the 50% probability level.

The title compound, C_15_H_11_ClOS, adopts a non-planar structure (Fig. 1 ▸) defined by the dihedral angle between the mean planes of the C1–C6 and C10–C16 phenyl rings of 13.06 (6)°. The major twist in the mol­ecule occurs about the C9—C10 bond [C8—C9—C10—C15 = −32.1 (2)°] and an intra­molecular O—H⋯S hydrogen bond (Table 1 ▸) supports the conformation. The bond lengths and bond angles are in good agreement with literature values for similar structures (Ganesha *et al.* 2023 ▸; Sreenatha *et al.* 2018 ▸, 2021 ▸; Nizamuddin *et al.* 2025 ▸; Lakshminarayana *et al.* 2022 ▸).

**Figure 2:**
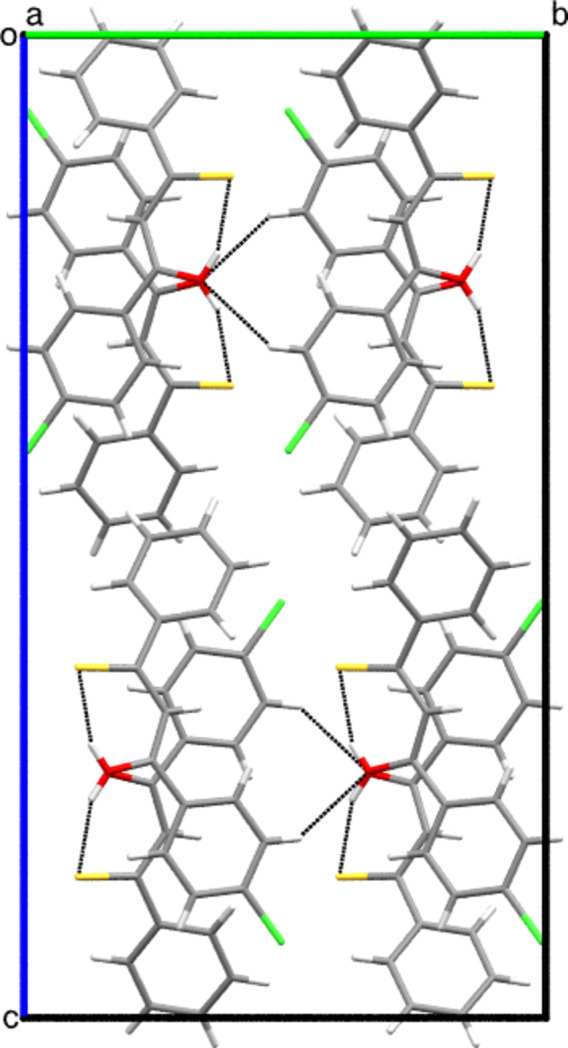
Packing of the title compound viewed along [100] with hydrogen bonds indicated by black dashed lines.

In the extended structure, weak C—H⋯O hydrogen bonds connect the mol­ecules into *C*(6) [010] chains (Fig. 2 ▸) with adjacent mol­ecules related by glide symmetry.

## Synthesis and crystallization

**Figure 3:**
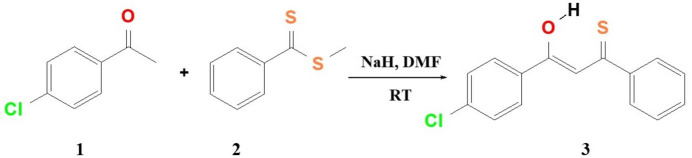
Synthesis scheme for the title compound.

To a stirred suspension of NaH (0.175 g, 2 eq.) in 20 ml of *N*,*N*-di­methyl­formamide (DMF) was added 4-chloro­acetophenon (1.00 g, 1 eq.) dropwise and the suspension was stirred at room temperature (Fig. 3 ▸). After 1 h, phenyl­dithio­ester (0.672 g, 1.1 eq.) dissolved in 20 ml of DMF was added dropwise and the stirring was continued at room temperature. After completion of the reaction (monitored by TLC), it was quenched with saturated aqueous NH_4_OH solution (50 ml) extracted with EtOAc (3 × 25 ml), washed with water (3 × 25 ml), brine (25 ml), and dried over anhydrous Na_2_SO_4_. The combined organic layer was evaporated under vacuum to give the title compound (single spot on TLC), which was passed through a silica gel column for further purification using hexa­ne/EtOAc (9.5:0.5), as eluent. Then, 0.5 g of the title compound was dissolved in 10 ml of DMF and was allowed to undergo slow evaporation (Nagaraju *et al.* 2020 ▸). Good quality crystals obtained were isolated and subjected to single-crystal X-ray diffraction studies.

## Refinement

Crystal data and structure refinement details are summarized in Table 2 ▸.

## Supplementary Material

Crystal structure: contains datablock(s) global, I. DOI: 10.1107/S2414314626005638/hb4563sup1.cif

Structure factors: contains datablock(s) I. DOI: 10.1107/S2414314626005638/hb4563Isup3.hkl

Supporting information file. DOI: 10.1107/S2414314626005638/hb4563Isup3.cml

CCDC reference: 2500235

Additional supporting information:  crystallographic information; 3D view; checkCIF report

## Figures and Tables

**Table 1:** Hydrogen-bond geometry (Å, °)

*D*—H⋯*A*	*D*—H	H⋯*A*	*D*⋯*A*	*D*—H⋯*A*
O1—H1⋯S1	0.82	2.11	2.8763 (12)	155
C6—H6⋯O1^i^	0.93	2.59	3.379 (2)	143

Symmetry code: (i) 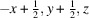.

**Table 2:** Experimental details

Crystal data	
Chemical formula	C_15_H_11_ClOS
*M*_r_	274.75
Crystal system, space group	Orthorhombic, *Pbca*
Temperature (K)	302
*a*, *b*, *c* (Å)	7.2296 (2), 13.8519 (3), 26.0849 (7)
*V* (Å^3^)	2612.24 (12)
*Z*	8
Radiation type	Mo *K*α
μ (mm^−1^)	0.44
Crystal size (mm)	0.28 × 0.25 × 0.23

Data collection	
Diffractometer	Bruker SMART CCD
No. of measured, independent and observed [*I* > 2σ(*I*)] reflections	90193, 3984, 3197
*R*_int_	0.046
(sin θ/λ)_max_ (Å^−1^)	0.715

Refinement	
*R*[*F*^2^ > 2σ(*F*^2^)], *wR*(*F*^2^), *S*	0.042, 0.136, 1.04
No. of reflections	3984
No. of parameters	163
H-atom treatment	H-atom parameters constrained
Δρ_max_, Δρ_min_ (e Å^−3^)	0.27, −0.38

Computer programs: *APEX* and *SAINT* (Bruker, 2012 ▸), *SHELXT2014/4* (Sheldrick, 2015*a* ▸), *SHELXL2019/2* (Sheldrick, 2015*b* ▸) and *PLATON*(Spek, 2020 ▸).

## full crystallographic data

### 3-(4-Chlorophenyl)-3-hydroxy-1-phenylprop-2-ene-1-thione Crystal data

**Table:**

C_15_H_11_ClOS	*D*_x_ = 1.397 Mg m^−^^3^
*M**_r_* = 274.75	Mo *K*α radiation, λ = 0.71073 Å
Orthorhombic, *P**b**c**a*	Cell parameters from 90193 reflections
*a* = 7.2296 (2) Å	θ = 3.0–30.5°
*b* = 13.8519 (3) Å	µ = 0.44 mm^−^^1^
*c* = 26.0849 (7) Å	*T* = 302 K
*V* = 2612.24 (12) Å^3^	Block, colorless
*Z* = 8	0.28 × 0.25 × 0.23 mm
*F*(000) = 1136	

### 3-(4-Chlorophenyl)-3-hydroxy-1-phenylprop-2-ene-1-thione Data collection

**Table:**

Bruker SMART CCD diffractometer	*R*_int_ = 0.046
Radiation source: graphite	θ_max_ = 30.5°, θ_min_ = 3.0°
ω scans	*h* = −10→10
90193 measured reflections	*k* = −19→19
3984 independent reflections	*l* = −36→36
3197 reflections with *I* > 2σ(*I*)	

### 3-(4-Chlorophenyl)-3-hydroxy-1-phenylprop-2-ene-1-thione Refinement

**Table:**

Refinement on *F*^2^	Primary atom site location: dual
Least-squares matrix: full	Hydrogen site location: inferred from neighbouring sites
*R*[*F*^2^ > 2σ(*F*^2^)] = 0.042	H-atom parameters constrained
*wR*(*F*^2^) = 0.136	*w* = 1/[σ^2^(*F*_o_^2^) + (0.0742*P*)^2^ + 0.5775*P*] where *P* = (*F*_o_^2^ + 2*F*_c_^2^)/3
*S* = 1.04	(Δ/σ)_max_ = 0.001
3984 reflections	Δρ_max_ = 0.27 e Å^−^^3^
163 parameters	Δρ_min_ = −0.38 e Å^−^^3^
0 restraints	

### 3-(4-Chlorophenyl)-3-hydroxy-1-phenylprop-2-ene-1-thione Special details

**Table:**

Geometry. All esds (except the esd in the dihedral angle between two l.s. planes) are estimated using the full covariance matrix. The cell esds are taken into account individually in the estimation of esds in distances, angles and torsion angles; correlations between esds in cell parameters are only used when they are defined by crystal symmetry. An approximate (isotropic) treatment of cell esds is used for estimating esds involving l.s. planes.
Refinement. Hydrogen atoms were placed in ideal positions and refined as riding model of C—H = 0.93 Å with *U*_iso_ (H) = 1.2*U*_eq_ (C) for aromatic H atoms.

### 3-(4-Chlorophenyl)-3-hydroxy-1-phenylprop-2-ene-1-thione Fractional atomic coordinates and isotropic or equivalent isotropic displacement parameters (Å^2^)

**Table:**

	*x*	*y*	*z*	*U*_iso_*/*U*_eq_	
S1	0.13146 (8)	0.10428 (3)	0.64334 (2)	0.05992 (16)	
Cl1	0.32731 (8)	0.49282 (4)	0.92234 (2)	0.06749 (17)	
O1	0.12163 (18)	0.16132 (8)	0.74933 (4)	0.0540 (3)	
H1	0.108232	0.129925	0.722935	0.081*	
C4	0.2303 (2)	0.30758 (10)	0.78307 (5)	0.0388 (3)	
C10	0.28603 (18)	0.25950 (10)	0.59518 (5)	0.0376 (3)	
C8	0.2416 (2)	0.27248 (10)	0.68877 (5)	0.0395 (3)	
H8	0.290559	0.334080	0.684611	0.047*	
C3	0.2164 (2)	0.26910 (11)	0.83215 (5)	0.0447 (3)	
H3	0.186004	0.204298	0.836171	0.054*	
C15	0.2767 (2)	0.35871 (11)	0.58661 (5)	0.0432 (3)	
H15	0.227176	0.398827	0.611690	0.052*	
C9	0.2216 (2)	0.21652 (10)	0.64414 (5)	0.0393 (3)	
C2	0.2469 (2)	0.32550 (12)	0.87512 (6)	0.0486 (3)	
H2	0.237457	0.299027	0.907778	0.058*	
C7	0.19601 (19)	0.24538 (10)	0.73824 (5)	0.0396 (3)	
C11	0.3609 (2)	0.20103 (12)	0.55686 (6)	0.0455 (3)	
H11	0.366388	0.134537	0.561551	0.055*	
C14	0.3403 (2)	0.39815 (12)	0.54114 (6)	0.0514 (4)	
H14	0.331563	0.464328	0.535660	0.062*	
C1	0.2911 (2)	0.42091 (12)	0.86880 (6)	0.0464 (3)	
C5	0.2752 (3)	0.40477 (11)	0.77817 (6)	0.0576 (4)	
H5	0.284178	0.432015	0.745679	0.069*	
C12	0.4271 (2)	0.24143 (13)	0.51187 (6)	0.0521 (4)	
H12	0.478848	0.201929	0.486868	0.063*	
C13	0.4169 (2)	0.33946 (14)	0.50382 (6)	0.0543 (4)	
H13	0.461181	0.366107	0.473488	0.065*	
C6	0.3067 (3)	0.46137 (12)	0.82081 (7)	0.0624 (5)	
H6	0.338160	0.526109	0.817167	0.075*	

### 3-(4-Chlorophenyl)-3-hydroxy-1-phenylprop-2-ene-1-thione Atomic displacement parameters (Å^2^)

**Table:**

	*U*^11^	*U*^22^	*U*^33^	*U*^12^	*U*^13^	*U*^23^
S1	0.0890 (3)	0.0388 (2)	0.0519 (3)	−0.01420 (19)	0.0021 (2)	−0.00697 (16)
Cl1	0.0811 (3)	0.0675 (3)	0.0538 (3)	−0.0044 (2)	0.0007 (2)	−0.0245 (2)
O1	0.0793 (8)	0.0396 (5)	0.0432 (5)	−0.0116 (5)	0.0043 (5)	0.0008 (4)
C4	0.0436 (7)	0.0361 (6)	0.0367 (6)	0.0021 (5)	0.0032 (5)	−0.0007 (5)
C10	0.0393 (6)	0.0396 (7)	0.0340 (6)	0.0018 (5)	−0.0037 (5)	−0.0045 (5)
C8	0.0467 (7)	0.0349 (6)	0.0370 (6)	−0.0030 (5)	0.0006 (5)	−0.0018 (5)
C3	0.0541 (8)	0.0412 (7)	0.0388 (7)	−0.0064 (6)	0.0012 (6)	0.0024 (6)
C15	0.0520 (7)	0.0408 (7)	0.0369 (7)	0.0019 (6)	−0.0002 (6)	−0.0036 (5)
C9	0.0430 (7)	0.0361 (6)	0.0387 (7)	0.0015 (5)	−0.0025 (5)	−0.0022 (5)
C2	0.0561 (8)	0.0537 (8)	0.0362 (7)	−0.0054 (7)	0.0015 (6)	0.0004 (6)
C7	0.0445 (7)	0.0356 (6)	0.0387 (7)	0.0012 (5)	0.0004 (5)	−0.0006 (5)
C11	0.0486 (7)	0.0447 (7)	0.0432 (7)	0.0080 (6)	−0.0015 (6)	−0.0083 (6)
C14	0.0619 (9)	0.0472 (8)	0.0450 (8)	−0.0011 (7)	−0.0003 (7)	0.0054 (6)
C1	0.0498 (8)	0.0467 (8)	0.0427 (7)	0.0011 (6)	0.0032 (6)	−0.0104 (6)
C5	0.0960 (13)	0.0371 (7)	0.0396 (7)	−0.0016 (8)	0.0063 (8)	0.0023 (6)
C12	0.0502 (8)	0.0660 (10)	0.0401 (7)	0.0089 (7)	0.0032 (6)	−0.0100 (7)
C13	0.0554 (9)	0.0685 (11)	0.0390 (7)	−0.0003 (8)	0.0043 (6)	0.0041 (7)
C6	0.1009 (14)	0.0359 (7)	0.0503 (9)	−0.0051 (8)	0.0081 (9)	−0.0048 (7)

### 3-(4-Chlorophenyl)-3-hydroxy-1-phenylprop-2-ene-1-thione Geometric parameters (Å, º)

**Table:**

S1—C9	1.6860 (15)	C15—C14	1.384 (2)
Cl1—C1	1.7353 (15)	C15—H15	0.9300
O1—C7	1.3148 (17)	C2—C1	1.370 (2)
O1—H1	0.8200	C2—H2	0.9300
C4—C5	1.391 (2)	C11—C12	1.385 (2)
C4—C3	1.3904 (19)	C11—H11	0.9300
C4—C7	1.4736 (18)	C14—C13	1.384 (2)
C10—C15	1.3940 (19)	C14—H14	0.9300
C10—C11	1.3959 (19)	C1—C6	1.376 (2)
C10—C9	1.4841 (19)	C5—C6	1.380 (2)
C8—C7	1.3837 (19)	C5—H5	0.9300
C8—C9	1.4060 (18)	C12—C13	1.376 (3)
C8—H8	0.9300	C12—H12	0.9300
C3—C2	1.384 (2)	C13—H13	0.9300
C3—H3	0.9300	C6—H6	0.9300
			
C7—O1—H1	109.5	O1—C7—C4	114.33 (12)
C5—C4—C3	118.21 (13)	C8—C7—C4	122.77 (12)
C5—C4—C7	122.16 (13)	C12—C11—C10	120.41 (15)
C3—C4—C7	119.63 (12)	C12—C11—H11	119.8
C15—C10—C11	118.42 (14)	C10—C11—H11	119.8
C15—C10—C9	121.22 (12)	C13—C14—C15	120.25 (16)
C11—C10—C9	120.34 (13)	C13—C14—H14	119.9
C7—C8—C9	126.72 (13)	C15—C14—H14	119.9
C7—C8—H8	116.6	C2—C1—C6	121.43 (14)
C9—C8—H8	116.6	C2—C1—Cl1	119.48 (12)
C2—C3—C4	121.18 (14)	C6—C1—Cl1	119.08 (13)
C2—C3—H3	119.4	C6—C5—C4	120.96 (15)
C4—C3—H3	119.4	C6—C5—H5	119.5
C14—C15—C10	120.68 (14)	C4—C5—H5	119.5
C14—C15—H15	119.7	C13—C12—C11	120.63 (14)
C10—C15—H15	119.7	C13—C12—H12	119.7
C8—C9—C10	117.33 (12)	C11—C12—H12	119.7
C8—C9—S1	123.95 (11)	C12—C13—C14	119.59 (15)
C10—C9—S1	118.72 (10)	C12—C13—H13	120.2
C1—C2—C3	118.97 (14)	C14—C13—H13	120.2
C1—C2—H2	120.5	C1—C6—C5	119.24 (15)
C3—C2—H2	120.5	C1—C6—H6	120.4
O1—C7—C8	122.88 (13)	C5—C6—H6	120.4
			
C5—C4—C3—C2	−0.2 (2)	C5—C4—C7—C8	13.5 (2)
C7—C4—C3—C2	−179.73 (15)	C3—C4—C7—C8	−167.04 (14)
C11—C10—C15—C14	0.0 (2)	C15—C10—C11—C12	1.1 (2)
C9—C10—C15—C14	178.81 (14)	C9—C10—C11—C12	−177.71 (14)
C7—C8—C9—C10	−177.99 (13)	C10—C15—C14—C13	−1.0 (2)
C7—C8—C9—S1	1.4 (2)	C3—C2—C1—C6	−0.4 (3)
C15—C10—C9—C8	−32.1 (2)	C3—C2—C1—Cl1	179.30 (13)
C11—C10—C9—C8	146.74 (14)	C3—C4—C5—C6	0.5 (3)
C15—C10—C9—S1	148.47 (12)	C7—C4—C5—C6	−179.97 (17)
C11—C10—C9—S1	−32.71 (18)	C10—C11—C12—C13	−1.2 (2)
C4—C3—C2—C1	0.1 (2)	C11—C12—C13—C14	0.2 (3)
C9—C8—C7—O1	−1.7 (2)	C15—C14—C13—C12	0.9 (3)
C9—C8—C7—C4	177.02 (14)	C2—C1—C6—C5	0.7 (3)
C5—C4—C7—O1	−167.72 (15)	Cl1—C1—C6—C5	−178.99 (16)
C3—C4—C7—O1	11.8 (2)	C4—C5—C6—C1	−0.8 (3)

### 3-(4-Chlorophenyl)-3-hydroxy-1-phenylprop-2-ene-1-thione Hydrogen-bond geometry (Å, º)

**Table:**

*D*—H···*A*	*D*—H	H···*A*	*D*···*A*	*D*—H···*A*
O1—H1···S1	0.82	2.11	2.8763 (12)	155
C6—H6···O1^i^	0.93	2.59	3.379 (2)	143

Symmetry code: (i) −*x*+1/2, *y*+1/2, *z*.
